# Knee Prosthesis in the Computer Era

**DOI:** 10.1111/os.12762

**Published:** 2021-01-27

**Authors:** Hasan B Sezer, Yoann Bohu, Alexandre Hardy, Nicolas Lefevre

**Affiliations:** ^1^ Clinique du Sport Paris V Paris France; ^2^ Institut de L'Appareil Locomoteur Nollet Paris France

**Keywords:** Computer assisted knee arthroplasty, Computer navigation, Literature review, Personalized cutting guides

## Abstract

Over the past two decades, computer assistance has revolutionalized surgery and has enabled enormous advancements in knee prosthesis surgery. Total knee arthroplasty (TKA) is a hot topic of orthopaedic research. Reflecting population dynamics, its use continues to increase, especially in high demand populations. Therefore, efforts to achieve the best fit and precise alignment in TKA continue. Computer assistance is valuable for knee prosthesis surgeons in this regard. This manuscript investigated the use of computer assistance in knee prosthesis surgery. The effects of computer use on important facets of knee prosthesis surgery, such as precision, clinical aspects, and costs, were examined. Moreover, an overall review of the recent literature on the navigation and personalized cutting guides was conducted.

## Graphical abstract

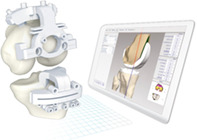

Computer assistance is a reasonable option to relieve the stress of the knee prosthesis surgeons. This manuscript provides a wide survey on the computer assistance in knee prosthesis surgery.

## Introduction

Total knee arthroplasty (TKA) is the gold standard for the treatment of severe knee joint degeneration^1^. TKA is highly effective in the treatment of knee osteoarthritis and patient satisfaction is high^1^, ^2^; therefore, the numbers of TKA in practice will inevitably continue to increase. The demand for primary TKA is expected to grow by 673% by 2030 in USA, with an estimated increase in revision rates between 78% and 182%^3^, ^4^. There is considerable interest in the TKA research to simulate normal knee performance and the accuracy of implantation, and in how TKA fulfills the needs of young patients with high functional demand, in a long‐living population.

The early designs of TKA were widely accepted, with their potential to decrease pain in osteoarthritic patients and improve walking ability compared to preoperative conditions. Early prosthetic technology, however, did not adequately simulate knee kinematics, leading to a lower return to sport rate after TKA compared to total hip arthroplasty and considerable revision rates due to polyethylene wear^5^, ^6^. As a reflection of the population dynamics, TKA patients are getting younger, are heavier, and are more active^7^. Thus, innovative efforts in orthopaedics have been concentrated on finding the best solution to increase the harmony of metallic and plastic components of TKA to increase function, as well as durability and longevity^8^. New TKA designs have led to remarkable improvement in clinical results and patient satisfaction. Snyder *et al*. found comparable patient reported outcomes for total hip arthroplasty patients and TKA patients receiving an optimized implant^9^.

Although the history of the evolution of TKA designs is not devoid of theoretically perfect but clinically unsustainable examples^10^, ^11^, ^12^, research continues to advance TKA. Implant design and materials, cementation, and postoperative knee alignment have been studied extensively^13^. The advances in polyethylene technology were a real breakthrough in the evolution of TKA, which decreased the revision rates significantly but did not eliminate the need for revision completely. Further improvement in polyethylene wear was demonstrated by either blending highly cross‐linked polyethylene with vitamin E or treating it with electron beam‐irradiation^8^, ^14^. The increased durability of polyethylene components prompted the idea of polyethylene tibial components to decrease costs^15^. Recently introduced in the market are bicruciate retaining or substituting and medial congruent prosthetic designs, which are still under investigation awaiting evidence of their long‐term survival performances to be favored in the clinical setting^16^, ^17^, ^18^, ^19^.

Computer‐assisted (CAS) knee prosthesis surgery is an evolving technique. Although recent improvements in materials and design have had a minor additive clinical effect, computer assistance has made enormous advancements both theoretically and practically two decades. This study was conducted to provide an up‐to‐date and thorough review of computer navigation systems and personalized cutting guides in knee prosthesis surgery. Different types of CAS knee prosthesis surgeries were compared in terms of precision, clinical aspects, and costs.

## Alignment and Balance

The current experience in TKA surgery is sufficient to demonstrate the importance of alignment and balance in the prevention of early polyethylene wear and prosthetic loosening^20^. To restore normal weight distribution of the joint, reconstruction of alignment is essential. There is a narrow safe range of deviation in the classical mechanical axis concept. A varus deformity of 3° is generally accepted as the limit of outliers^21^, ^22^. Conversely, the kinematic alignment concept emerged as a new notion in TKA based on previous personal knee alignment instead of mechanical alignment to maximize ligamentous balance^23^. It is assumed to be more anatomical than the mechanical alignment concept and to simulate the knee ligamentous synergy better than the conventional method^24^, although the clinical impact is questionable^24^, ^25^.

Introduced in the early 1990s, CAS‐TKA has been a topic of interest in orthopaedic research^26^. Efforts to achieve the best fit and a precise alignment influenced orthopaedists to use computer technology^27^. Digital technology made three‐dimensional (3D) planning possible, which allowed health‐care providers to better understand the complex anatomy of the knee and to improve accuracy in theatre compared to the conventional technique^28^. The *in‐situ* advantage of computer technology emerged with its intraoperative use to guide component positioning. As in most fields of science, computer assistance in orthopaedics decreases capacity for human error and improves outcomes. A report on the Australian national joint registry investigated the long‐term survival of 44,573 computer navigated primary joint arthroplasties and revealed remarkably better coronal alignment and decreased revision TKA operations due to aseptic loosening with computer assistance compared to conventional TKA procedures^29^. The basic function of CAS systems is their assistance to surgeons through linking medical images with the real anatomy^30^.

## Computer‐Assisted Total Knee Arthroplasty Systems

Presently, there are two main categories of CAS‐TKA systems in the market. TKA with computer navigation (CN) synchronizes preoperative and intraoperative measurements with information gathered by intraoperative visualization, to control the implant positions, *in vivo*. In contrast, patient‐specific instrumentation (PSI) uses cutting guides produced *in vitro*, based on preoperative either CT or magnetic resonance images of the patient, to help the operator in deciding the optimal prosthetic orientation while in the operating room.

**Fig 1:**
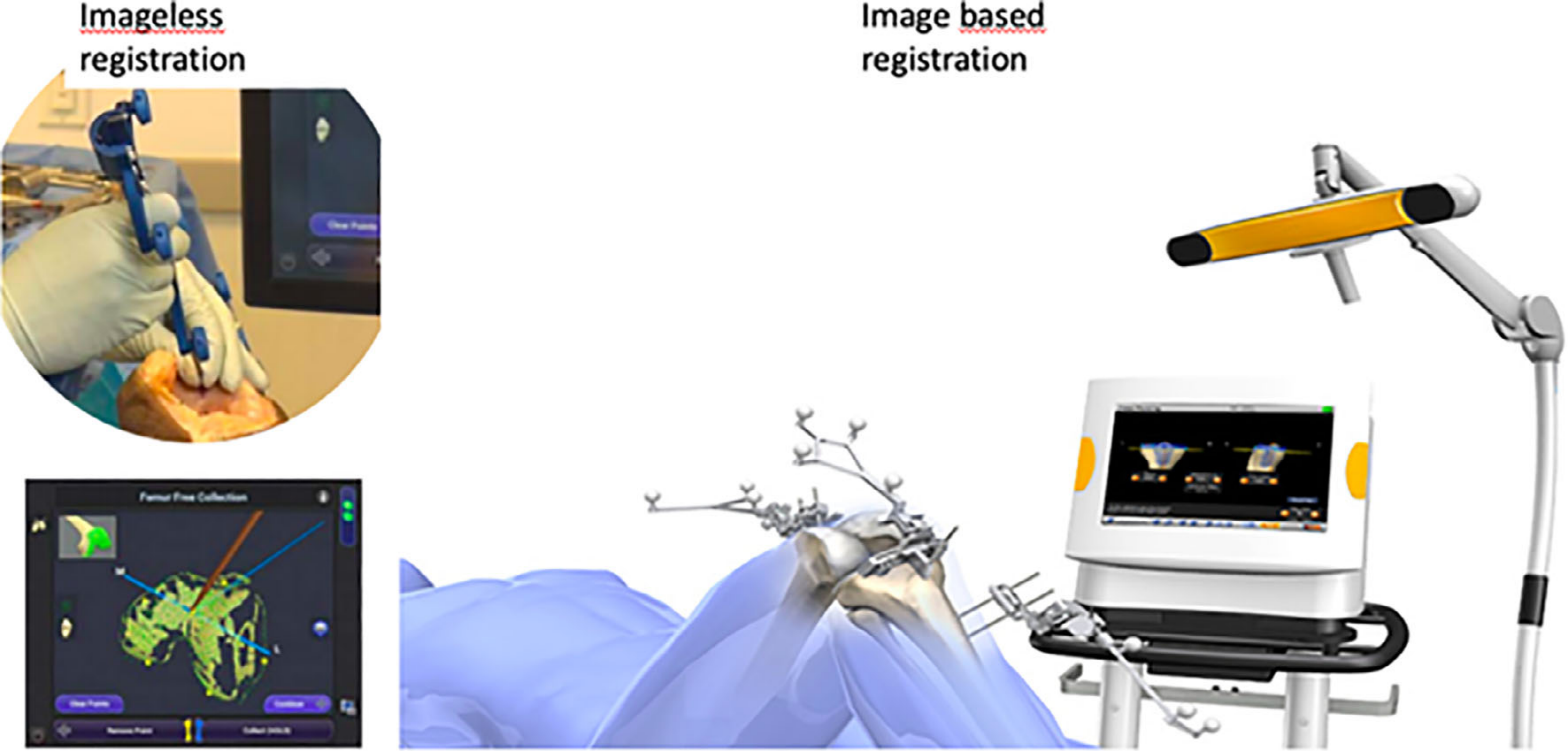
Imageless and image‐based registration of anatomical landmarks.

Computer navigation systems may be roughly classified into three main categories: image‐based large console navigation, imageless large console navigation, and hand‐held systems^31^. All of these systems require landmarks like the center of the hip or the ankle, and various other landmarks around the knee joint, to enable coordination between the computer and the cutting instruments. Image‐based systems use either CT or fluoroscopy. Imageless CAS, also called morphing surface navigation, uses preregistered data on the general population for normalization of intraoperative morphologic data required from the patient to guide the position of cutting blocks (Fig. 1)^32^.

The first designs of CAS units suggested pin placement in the pelvic bone to help determine the femoral head position, which was abandoned with the kinematic determination of the hip joint position^32^. The information on landmarks is transferred to be processed by the console, which produces feedback to the surgeons. Bone cuts are made either freehand or with robotic assistance^30^. The CN systems help surgeons with intraoperative precision, especially in hard cases. Periarticular bone deformities and implants are good candidates for CAS‐TKA^14^.

**Fig 2:**
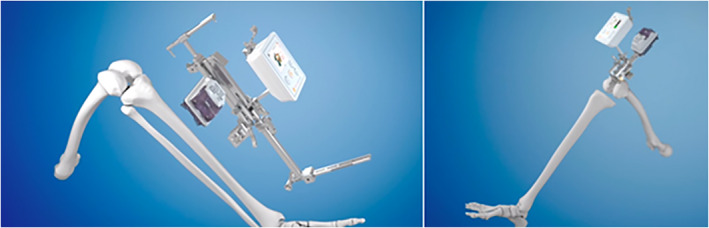
Accelerometer‐based registration.

Handheld navigation systems are mainly accelerometer‐based systems. They are remarkably low cost compared to their counterparts (Fig. 2). Budhiparama *et al*. (2019) published a review article on handheld navigation systems, including 11 manuscripts and 1298 TKA patients, to compare their accuracy, functional outcomes, and surgical times with the conventional technique^33^. However, there was no evidence to conclude that there was an improvement in clinical or patient‐reported outcome measures with handheld systems, but increased expense was demonstrated, with the longer operative times.

In 1998, Radermacher *et al*. presented a new technique utilizing 3D printing technology, PSI^34^. PSI is basically a method involving custommade instrument guidance for bone preparation in TKA. The cutting blocks are printed in the laboratory based on patients' preoperative medical images. A 3D model of a patient's knee is produced, as well, to check the accordance of the guides to the patient's knee. When applying this technique, it is possible to use a standard or personalized prosthesis (patient‐specific arthroplasty) according to surgeon preference^35^.

## Patient‐Specific Instrumentation: Preoperative Imaging and Planning

The process of PSI begins with the evaluation of 3D images, either magnetic resonance images or computerized tomography (CT) images, and long leg standing X‐rays to assist in digitally restoring the anatomy of the patient. It is important to provide hip and ankle slices, as well, to correctly delineate the mechanical axis of the lower extremity. The accuracy of the preoperative plan is key for the efficiency of PSI. To decrease the risk of intraoperative changes, the participation of the orthopaedic surgeon in the preoperative planning of PSI is indispensable (and should not be left to manufacturers)^36^.

Computed tomography is faster to acquire and is cheaper than magnetic resonance imaging (MRI) at the cost of radiation exposure. CT is the choice of modality in the presence of metallic implants. MRI‐based patient‐specific cutting guides are reported to produce greater accuracy in terms of coronal alignment compared to CT^37^. However, controversy exists in regard to femoral and tibial component alignment with the utilization of CT or MRI^38^, ^39^. From a technical aspect, MRI‐based guides are produced considering the presence of cartilage. In contrast, when using a CT‐based guide, the cartilage is to be thoroughly removed.

**Fig 3:**
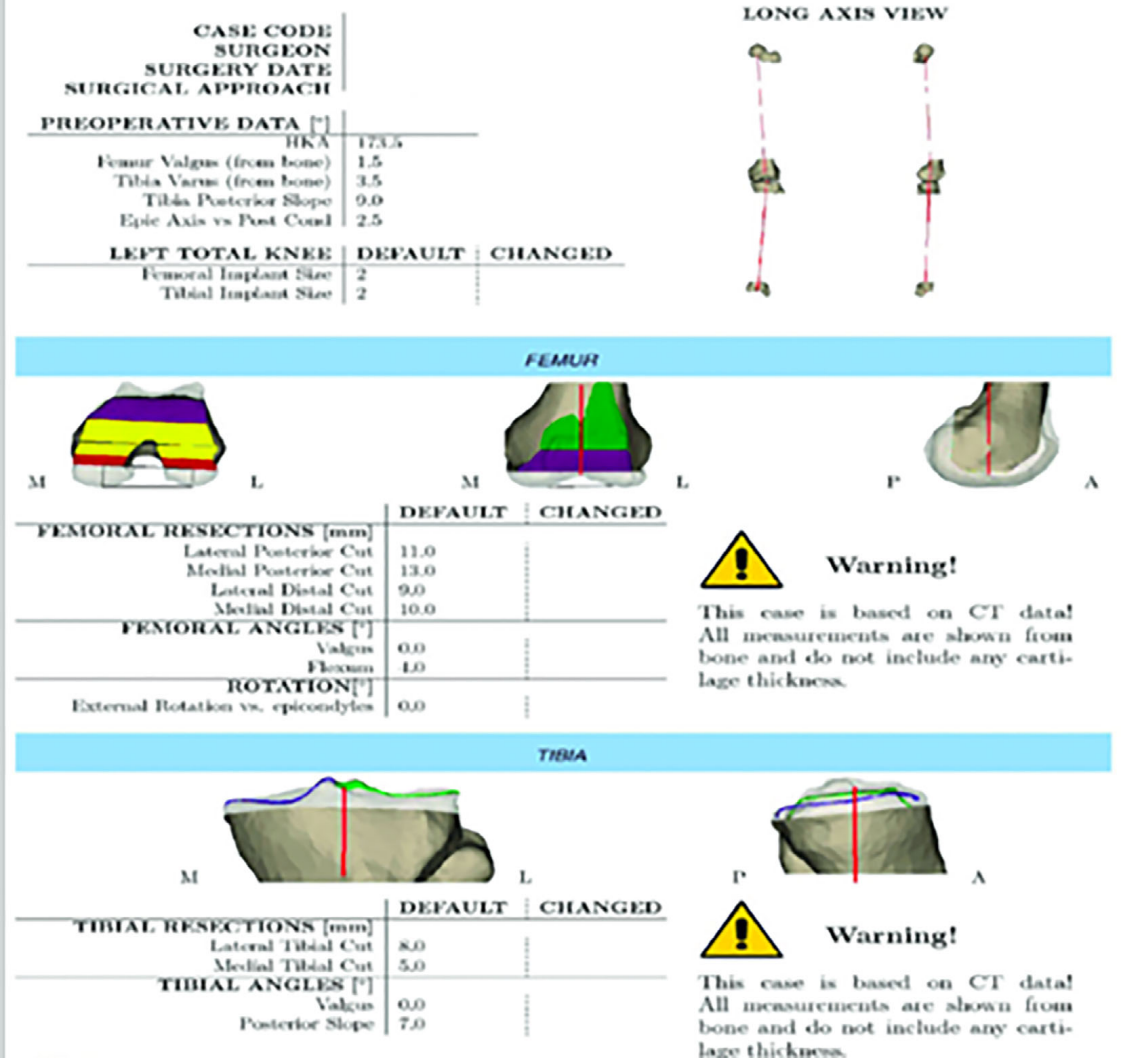
An example of computed tomography‐based preoperative planning.

In the following step, using the PSI planning software, the size and type of the implants are determined. PSI is accurate in predicting prosthesis sizes preoperatively^40^. The 3D knee model and the cutting guides are used to determine the precise femoral and tibial cuts. There exist distinct anatomical characteristics, such as bony prominences or clefts in each bone, which serve as landmarks to match and secure the guide. The ability to try the matching guides with bone models gives the surgeon the opportunity to understand their relation with real bone and the direction and magnitude of bone cuts when placing the printed guides on the real bone before implementation (Fig. 3).

## Clinical Results of Computer‐Assisted Knee Prosthesis Surgery Systems

Prosthetic malalignment, especially coronal outliers, is regarded as a major failure risk for TKA^20^. The real drive behind the idea of CAS‐TKA is perfection of prosthesis positions and alignment. Although there are studies that report better coronal component alignment using CAS compared to conventional TKA procedures^41^, ^42^, controversy exists^43^, ^44^. In a review study published by Mannan *et al*., six studies fulfilled inclusion criteria, with 444 TKA included. They found favorable femoral rotational alignment with PSI‐TKA but insufficient evidence concerning tibial component rotation^45^, ^46^. Heyse *et al*. conducted a study of 28 PSI and 30 conventional TKA patients with postoperative tibial component rotation and reported better rotational tibial component alignment in the PSI group^47^.

Unfortunately, none of those systems are foolproof and they require expertise to achieve a good radiological and functional result. In addition, comparable clinical and radiological outcomes with CAS systems were reported in the literature on conventional TKA. Recent high‐quality studies comparing conventional TKA and CAS‐TKA have reported many controversies regarding which technique yields better clinical and radiological results^47^, ^48^, ^49^, ^50^. However, the CAS‐TKA has already provided outcomes equivalent to those of experienced surgeons using traditional TKA, with the potential for improvements with further technological advances^51^. In comparing first and second generations of PSI with the conventional TKA, the second generation of PSI is reported to perform better in terms of fit, improving both the alignment and decreasing surgical times^41^.

**Fig 4:**
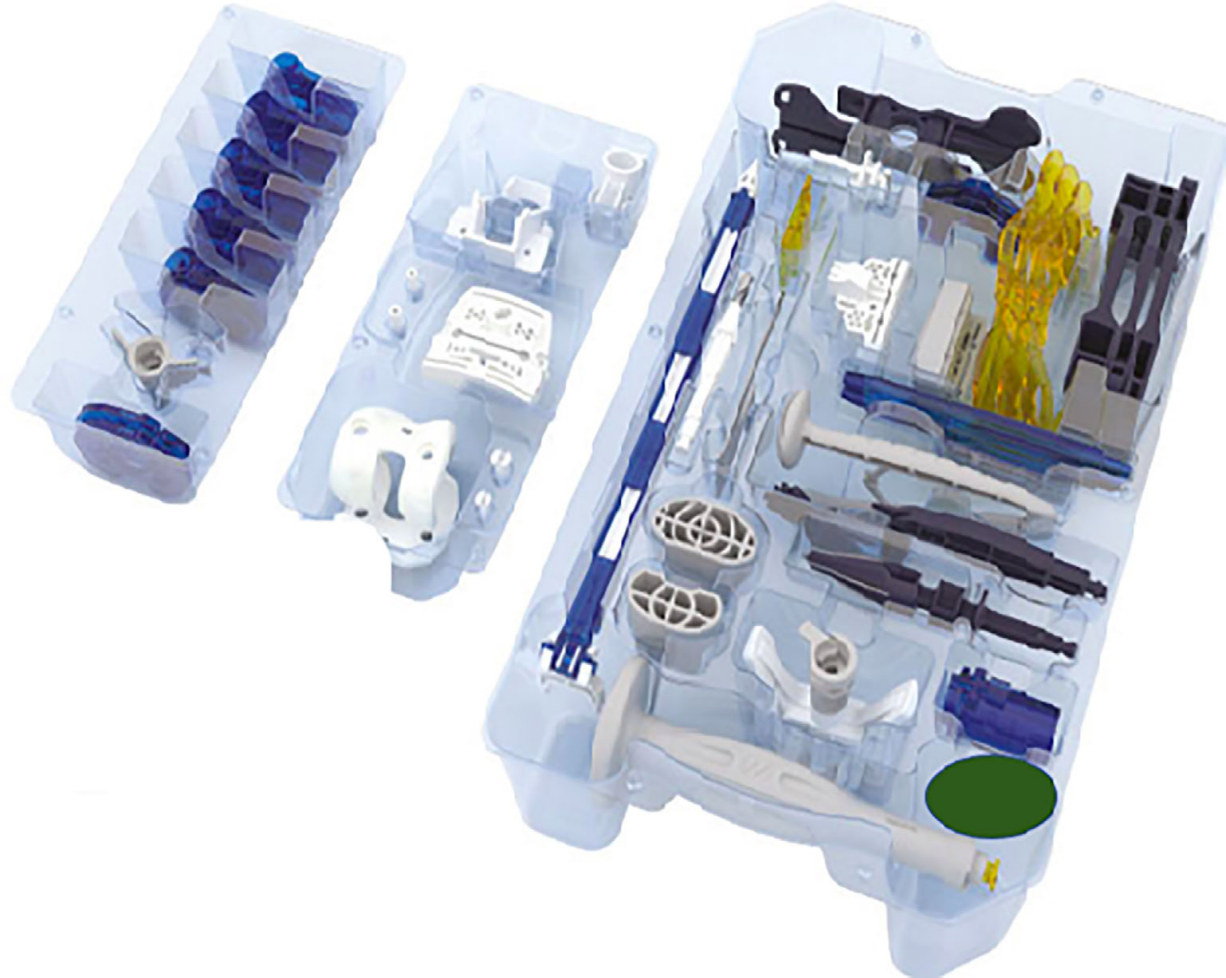
Demonstration of single‐use instrument trays and three‐dimensional cutting guides.

There are numerous studies on PSI to compare its efficacy radiologically and clinically with other techniques. However, there are not many studies that compare different PSI brands. Lin *et al*. (2020) published a review that reported better alignment with one of the PSI models. High‐quality comparative studies on different PSI models are needed to highlight the different technological aspects of each PSI model and their impact on radiological and clinical results. PSI is assumed to be associated with decreased blood loss, which eliminates the need to open the femoral intramedullary canal^48^, ^52^. Thienpont *et al*. conducted a study that included 75 PSI‐TKA patients, to determine if there was any influence of PSI on blood loss. The authors report no significant difference between PSI and conventional TKA^53^. However, other researchers report decreased blood loss with PSI compared to the conventional method and CAS‐TKA^54^, ^55^. Increased blood loss in TKA is reported to be related to increased risk of infection^56^, ^57^. The blood loss with PSI is reported to be equal to or lower than that of the conventional method or navigation systems, which indicates that PSI has the ability to lower infection risk. The instruments for PSI are made of plastic, are fewer in number, and are disposable; they are not heavy and there are no sharp metal instruments. These are important features that reduce the risk of non‐sterilization or impaired sterility compared to heavy conventional trays (Fig. 4).

## Operating‐Room Management and Costs

One of the possible advantages of the PSI method is better operation room management. PSI in TKA is simpler and faster than standard instrumentation methods^58^. In contrast to the complicated production process in the factory, the trays of PSI are not complex^59^. Renson *et al*. demonstrated improved alignment and operating room efficiency with PSI. The study was conducted with 71 PSI and 60 conventional TKA patients and revealed a reduction in surgical time of approximately 8.6 min and operation room time of 8.9 min^60^. The number of surgical trays was decreased dramatically with these single‐use instruments, by 6 to 7.3 trays for each TKA. Kwon *et al*. reported 18.5 min shorter operation time with new generation PSI^41^.

Additional costs of navigation systems are mainly the institutional costs required for CAS modules and preoperative 3D imaging, and those resulting from increased surgical and operating room times. Moreover, PSI systems are dependent on pre‐production of plastic models. However, decreased need for sterilization and better surgical room management may exert beneficial effects on the operational cost of PSI. Most of the previous studies have reported an increase in surgical time of 10 to 30 min for each operation with CAS‐TKA to finish landmark registration. In cases of improper registration, pin loosening, or fracture, all of the data will be unreliable^61^. Despite their powerful assistance, large console CN systems exerted a steep increase in utilization even in the United States, from 1.2% in 2005 to 6.3% in 2014 regarding the excessive hospital charges^62^. The computer navigated TKA stays still specific to surgeons who work in the facilities that hold one due to its cost, difficulties in implementation, and innate complications^63^. DeHaan *et al*. studied the costs and savings associated with PSI for TKA. The expenses for PSI, including preoperative imaging and the guide itself, were reported to be between $930 and $1860. However, the average cost saving for each case was calculated as $1566, which included the savings from operating room management and sterilization. The authors concluded that the imaging costs were institution‐dependent and had a wide range^64^.

## Conclusion

Consistent with previous studies, recent reviews revealed that the superiority of the CAS‐TKA technique over the conventional technique remains uncertain in the short and long term^65^. However, the efforts to better synchronize metal and plastic components to represent a normal knee continue to revealing minor but critical advances in precision. Modern medicine has led to decreased pain and mid‐term survivorship of TKA, whereas long‐term survivorship and performance of knee replacement still need to be improved.

The issue of alignment and balance continues to occupy the orthopaedic literature. Either anatomical or kinetic alignment concept is adopted into the daily practice, it has to be applied precisely. As the number of alignment studies increases either in favor of PSI or reporting no real advantage of PSI, there are no studies showing the inferiority of this technique. It is important to remember that whatever the technique used, the real decision‐maker in an operation room is the surgeon. Therefore, the technique used will be favored if it helps facilitate the procedure. The simplicity of the use of PSI in the field compared to other CAS systems may favor the acceptance of this technique in the future.

There is a growing involvement of computer technology in orthopaedic surgery.^66^ The use of computer assistance in TKA is an example of an initiative to augment human decision‐making and surgical handicraft with artificial intelligence. The advancement of computer assistance in orthopaedic surgery should be supported. The next step in CAS surgery may be realized in the future with the support of high‐level evidence supplied by clinical studies.
